# Impact of Cotadutide drug on patients with type 2 diabetes mellitus: a systematic review and meta-analysis

**DOI:** 10.1186/s12902-022-01031-5

**Published:** 2022-04-29

**Authors:** Mahmoud M. Ali, Ahmed Hafez, Mahmoud Shaban Abdelgalil, Mohammed Tarek Hasan, Mohammed Magdy El-Ghannam, Osama M. Ghogar, Asmaa Ahmed Elrashedy, Mohamed Abd-ElGawad

**Affiliations:** 1 Faculty of Pharmacy, Al-Azhar University, Assiut, Egypt; 2 Faculty of Medicine, Menoufia University, Menoufia, Egypt; 3 Faculty of Medicine, Ain-Shams University, Cairo, Egypt; 4 Faculty of Medicine, Al-Azhar University, Cairo, Egypt; 5 Faculty of Pharmacy, German University in Cairo, Cairo, Egypt; 6 Faculty of Medicine, Kafr El-Shaikh University, Kafr El-Shaikh, Egypt; 7 Faculty of Medicine, Fayoum University, Fayoum, Egypt

**Keywords:** Type 2 diabetes mellitus, Cotadutide, Glucagon-like peptide 1, Weight loss

## Abstract

**Background:**

The food and drug administration approved many drugs to treat diabetes mellitus, but those drugs do not have a noticeable effect on weight management. Recently, glucagon-like peptide 1 agonist known as Cotadutide serve as a potent drug in treating type 2 diabetes by reducing blood glucose levels and body weight indices. This study aimed to explore the safety and efficacy of Cotadutide as a treatment for type 2 diabetes individuals.

**Methods:**

A comprehensive literature search was done on different databases, including PubMed, Scopus, Web of Science, and Cochrane Library to capture all relevant articles using an established search strategy. The inclusion criteria were randomized controlled trials that assessed the safety and efficacy of Cotadutide versus placebo or any anti-diabetes drugs in patients with type 2 diabetes mellitus and a BMI between 22 kg/m^2^ and 40 kg/m^2^. We conducted the analysis using Revman software version 5.4.

**Results:**

We found 663 relevant articles. From which nine studies were included and subjected to qualitative analysis and eight for quantitative analysis. The pooled effect showed that Cotadutide was better than placebo in reducing body weight (kg) (Mean difference (MD) = 3.31, *p* < 0.00001), glycated hemoglobin (HbA_1c_) (MD = 0.68, *p* > 0.00001), glucose area under the plasma concentration curve (AUC [0-4 h]) (MD = 30.15, *p* < 0.00001), and fasting plasma glucose over time (mg/dl) (MD = 31.31, *p* < 0.00001).

**Conclusion:**

Cotadutide is safe and effective in reducing plasma glucose levels, HbA_1c_ and body weight in individuals with type 2 diabetes.

**Trial registration:**

The study protocol was registered on PROSPERO (CRD: CRD42021257670).

**Supplementary Information:**

The online version contains supplementary material available at 10.1186/s12902-022-01031-5.

## Background

Type 2 diabetes mellitus is one of the most common endocrine disorders worldwide, according to the International Diabetes Federation (IDF) its prevalence has surged rapidly to include more than 400 million individuals over the past three decades [1]. Type 2 diabetes mellitus is a long-term disease characterized by chronic insulin resistance and hyperglycemia that increases over time, resulting in increasing of insulin resistance leading to weight gain [2, 3]. Therefore, reducing body weight will prevent more insulin resistance and better control of the body weight condition.

Many medications with different mechanisms are available to control type 2 diabetes mellitus as (a) metformin which acts through various trajectories to inhibit gluconeogenesis and reduce the level of lipopolysaccharide, (b) insulin secretagogues, (c) alpha-glucosidase inhibitors, (d) dipeptidyl peptidase 4 inhibitors, and (e) sodium-glucose co-transporter-2 inhibitor [4]. However, none of them is significant in reducing body weight at doses approved for blood glucose reduction. Therefore, weight loss remains an unmet medical need for these people [5].

Glucagon-like peptide-1 (GLP-1) receptor agonist's therapy known as Cotadutide seems to be effective in glycemic control and weight loss. The impact of GLP-1 drugs varies depending on the pharmacokinetic profile [6]. Lorenz M et al. 2013 showed that short-acting GLP-1 receptor agonists (lixisenatide) at a dose of 20 μg daily lowers postprandial hyperglycemia excursions in individuals with type 2 diabetes mellitus, probably caused by the continuous slowing of stomach emptying [7]. In the same way, J van Can et al. 2014 investigated the effects of long-acting GLP-1 receptor agonists (liraglutide) on gastric emptying and the result indicated that liraglutide at 3 mg significantly delays the gastric emptying [8]. In addition, Daniel R et al. reported in 2020 that lixisenatide reduced the gastric emptying rate more than liraglutide [9].

Based on the above-mentioned data, GLP-1 receptor agonists are useful in treating individuals with type 2 diabetes and obesity by controlling hyperglycemia and delaying stomach emptying. They also help people lose weight by reducing the appetite and increasing energy expenditure by optimizing metabolic reactions such as amino acid catabolism, and fatty acid oxidation [10].

Many studies have investigated the effect of Cotadutide (GLP-1 receptor agonist) on type 2 diabetes mellitus. In this study, we aim to summarize, review, and analyze those studies to understand the safety and efficacy profiles of this new medication in controlling type 2 diabetes mellitus and its effect on weight reduction.

## Methods

### Study design and registration

This meta-analysis was conducted following the Preferred Reporting Items for Systematic Reviews and Meta-Analysis (PRISMA) guideline and Cochrane Handbook of Systematic Reviews of Intervention [11, 12]. The study protocol was registered on PROSPERO (CRD: CRD42021257670).

### Literature search

PubMed, Cochrane Central Register of Controlled Trials (CENTRAL), Scopus, and Web of Science were searched for articles conducted from 1 January 1979 to 1 June 2021 without any other restrictions. We used Mesh database to generate the search strategy used. The search strategy is formed of a combination of the following keywords and their relative words (Cotadutide) AND (Diabetes) AND (body weight). The detailed search strategy can be found in supplementary file 1.

### Eligibility criteria and studies selection

The inclusion criteria included Randomized Controlled Trials (RCT) evaluating the efficacy and safety of the drug Cotadutide on men or women aged 18 to 65 years with controlled type 2 diabetes and a BMI between 22 kg/m^2^ and 40 kg/m^2^. Only English studies were included which provide full text online accessible to us. No restrictions regarding the date of publication. Protocols published in clinicaltrials.gov were included if they contain results and sufficient information to assess their quality.

We excluded studies with insufficient data for extraction. Reviews, book chapters, thesis, editorial, letters, conference papers, and non-English studies. Animal or In vitro studies, cohort, case–control, non-clinical studies, literature reviews, and meta-analysis were excluded.

Two independent authors screened the articles retrieved from the four electronic databases by title, abstract, and full text on an excel sheet for eligibility. Another independent author resolved any disagreements between the other two authors.

### Quality assessment

Cochrane risk-of-bias tool for randomized trials (RoB 2) was applied to assess the quality of the selected RCTs [13]. The Rob2 tool consists of six domains: randomization process, deviations from the intended interventions, missing outcome data, measurement of the outcome, selection of the reported result, and other biases. The evaluators' responses were categorized as yes, probably yes, probably no, no, and no information. Following that, all disputes were discussed and resolved.

### Data extraction and study outcomes

Two independent auhrors extracted data in a pre-defined excel sheet. The excel sheet items were categorized as a summary of the included trials' key features, characteristics of the participants, and Cotadutide safety and efficacy outcomes. Any disagreements were solved by a discussion between the reviewers.

### Outcome definition

Treatment efficacy was assessed by frequency of positive Anti-drug antibodies to Cotadutide, Percent Change from Baseline in Body Weight, Change from Baseline in Glycated Hemoglobin (HbA_1c_), Mean Percentage Change from Baseline in Glucose Area Under the Plasma Concentration Curve (AUC [0-4 h]) as Measured by (MMTT). The safety outcomes included Treatment-Emergent Adverse Events (TEAEs), and Treatment-Emergent Serious Adverse Events (TESAEs).

### Data synthesis and assessment of heterogeneity

We performed all statistical analyses using Revman software Version 5.4.1. The present meta-analysis estimated the pooled risk ratio (RR) for dichotomous data, mean difference (MD) for continuous data with 95% confidence intervals (CI). The significance point was set at *p*-value less than 0.05.

We assessed the heterogeneity using the I-square and p-value. The analysis was considered heterogeneous if it had a *p*-value less than 0.05 or an I-square less than 50%. A random-effect model was applied if heterogeneity was detected and a leave one out test was performed to determine which study was causing the heterogeneity [14].

## Results

### Data collection and study selection

**Fig. 1:**
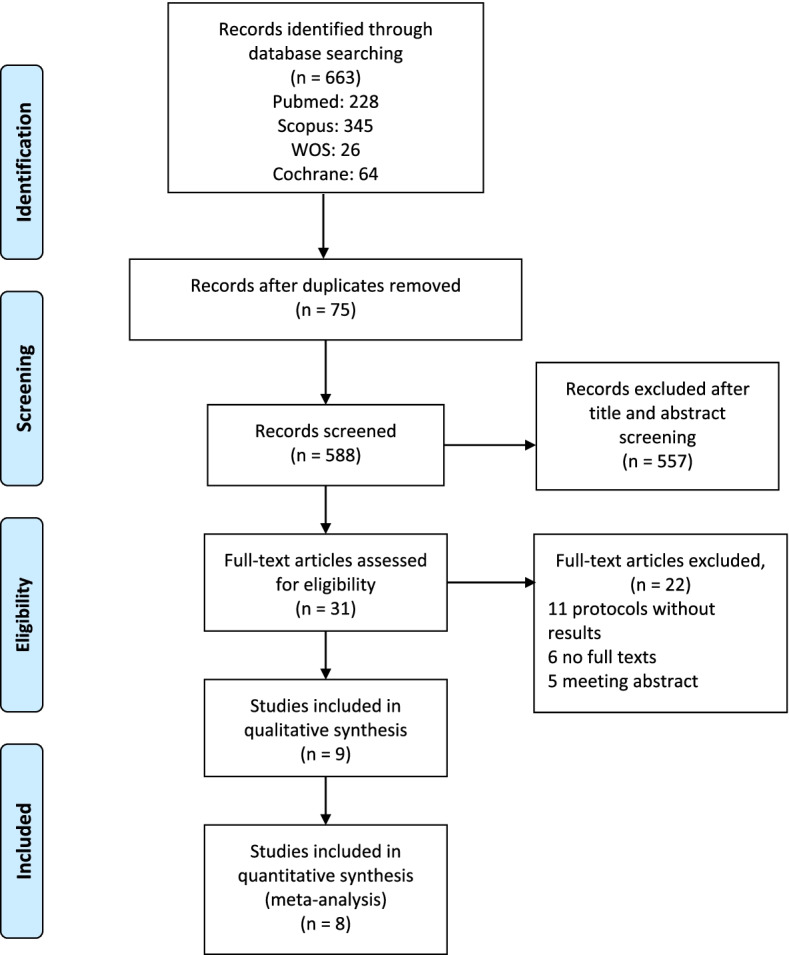
Preferred Reporting Items for Systematic Reviews and Meta-Analyses (PRISMA)

Our search retrieved 655 records from PubMed, Scopus, Web of Science, and Cochrane library. There were 75 duplicates. After title and abstract screening, we eliminated 557 records. Afterward, we screened 31 studies for eligibility, 22 studies were excluded. Eleven studies were protocols without results, six were without full texts available, and five were meeting abstracts. Finally, nine records were included in our study: four published clinical trials and five registered protocols from Clinicaltrials.gov, and only eight studies were included in the meta-analysis (Fig. 1).

The total sample size for this meta-analysis was 1259 participants (259 persons received a placebo, 890 participants received Cotadutide and 110 participants received other interventions). There were no concomitant treatment modalities except in two studies. In NCT03235050, participants in all study groups received metformin tablets and a separate group was treated with liraglutide to compare it with Cotadutide and placebo. Moreover, during the treatment period of the study NCT03444584, participants were on metformin and dapagliflozin as well. Table 1 elucidates the full summary of the included studies. The baseline characteristics of the participants are illustrated in Table 2.

**Table 1:** Summary of the included studies

Study ID	Title	Study design, country, and timing	Criteria	Sample size	MEDI0382 treatment regimen	Control group	study duration
P. Ambery et al. (2018) [6]	MEDI0382, a GLP-1 and glucagon receptor dual agonist, in obese or overweight patients with type 2 diabetes: a randomized, controlled, double-blind, ascending dose and phase 2a study	RCT Germany, Dec 9, 2015, and Feb 24, 2017	• Men or women aged 18–65 years with controlled T2DM • HbA1c levels of 6·5–8·5% at screening • BMI between 27 kg/m^2^ and 40 kg/m^2^ • Individuals who received metformin monotherapy of 500 mg or more within three months before screening or received an adjunct to metformin were eligible after a 4-week washout period	113 patients (68 for MEDI0382, and 45 for placebo)	MEDI0382 100 mcg *N* = 6 MEDI0382 150 mcg *N* = 6 MEDI0382 200 mcg *N* = 7 MEDI0382 200 mcg *N* = 25 MEDI0382 300 mcg *N* = 12 MEDI0382 300 mcg *N* = 12	*N* = 45 Participants received placebo matched to MEDI0382 dose	3 years
P. D. Ambery (2018) [15]	MEDI0382, a GLP-1/glucagon receptor dual agonist, meets safety and tolerability endpoints in a single-dose, healthy-subject, randomized, Phase 1 study	RCT Germany, 26 February 2015 and 12 August 2015,	• Healthy volunteers, male or female aged 18–45 years, are chosen approximately to a population of T2DM patients minimizing the chance for concomitant medical conditions • BMI between ≥ 22 kg.m–2 and ≤ 30 kg.m–2, • Bodyweight ≥ 70 kg • Individuals were required to have venous access suitable for multiple cannulations	48 patients (36 for MEDI0382, and 12 for placebo)	MEDI0382 5 μg N = 6 MEDI0382 10 μg N = 6 MEDI0382 30 μg N = 6 MEDI0382 100 μg N = 6 MEDI0382 300 μg N = 6 MEDI0382 150 μg N = 6	*N* = 12 Participants received placebo matched to MEDI0382 dose	3 years
Rajaa Nahra (2021) [16]	A Study to Evaluate the Efficacy and Safety of MEDI0382 in the Treatment of Overweight and Obese Subjects with Type 2 Diabetes	RCT, Bulgaria, Canada, Czechia, Germany, Mexico, Russian Federation, Slovakia, United States, 2 August 2017 and 14 June 2019	• Male and female subjects aged ≥ 18 years with T2DM • BMI ≥ 25 kg/m2 • HbA1c range of 7.0% to 10.5% • Individuals Treated with metformin ≥ 1500 mg/day or more for at least two months before screening or using adjuvant medication for up to 2 weeks in the two months before screening is acceptable	834 patients (612 for MEDI0382,110 for Liraglutide and 112 for placebo)	MEDI0382 100 mcg *N* = 100 MEDI0382 200 mcg *N* = 256 MEDI0382 300 mcg *N* = 256	*N* = 112 Participants received placebo / Metformin tablets, total daily dose of ≥ 1500 mg (unless only tolerated at a lower dose)	2 Years
NCT03745937	A Study to Evaluate the Safety and Tolerability of MEDI0382 in Overweight and Obese Subjects With Type 2 Diabetes Mellitus	RCT, Germany	• Participants aged 18 to 74 years with T2DM • BMI between 27 and 35 kg/m^2 • HbA1c range of 6.5% to 8.5% • Individuals Treated with metformin monotherapy where no significant dose change (increase or decrease > = 500 mg/day) has occurred in the three months before screening	20 patients (18 for MEDI0382 and 2 for placebo)	Participants received SC dose of MEDI0382 up-titrated weekly once daily up to 8 weeks during the up-titration period and thereafter once daily in 3-week TEP	*N* = 2 Participants received placebo matched to MEDI0382 dose	4.5 months
NCT03645421	Safety and Tolerability Study of MEDI0382 in Japanese Pre-obese or Obese Subjects With Type 2 Diabetes	RCT, Japan	• Individuals diagnosed with T2DM • HbA1c range of 7.0% to 10.5% • Individuals with drug naïve at Visit 1 • BMI within the range of 24—40 kg/m2	61 patients (45 for MEDI0382 and 16 for placebo)	MEDI0382 100 mcg *N* = 15 MEDI0382 200 mcg *N* = 15 MEDI0382 300 mcg *N* = 15	*N* = 16 Participants received placebo matched to MEDI0382 dose	5 months
NCT03596177	A Study to Evaluate the Effect of MEDI0382 on Energy Balance in Overweight and Obese Subjects With Type 2 Diabetes Mellitus	RCT, United Kingdom	• Participants aged > = 30 and < = 75 years with T2DM • Body Mass Index > 28 and < = 40 kg/m^2 • HbA1c < = 8.0% • Individuals treated with metformin, with or without another adjuvant drug (increase or decrease > 50%) occurred three months before screening • For the participant on dual therapy, a 4-week washout of the non-metformin therapy will be performed before Visit	28 patients (19for MEDI0382 and 9 for placebo)	MEDI0382 titrated up to 300 μg *N* = 19	*N* = 9 Participants received placebo matched to MEDI0382 dose	1 year and 1 month
NCT03444584	Study of MEDI0382 in Combination With Dapagliflozin and Metformin in Overweight/Obese Subject With Type 2 Diabetes	RCT, Germany, and Hungary	• Male and female participants aged > = 18 years with T2DM • BMI between 25 kg/m^2 and 40 kg/m^2 • HA1c ranges between 7.0% and 10.0% • Individuals treated with metformin monotherapy (MTD > 1 g) at least eight weeks or treated with stable, oral doses of dapagliflozin 10 mg and metformin (MTD > 1 g) for at least three months before screening • For the participant on dual therapy in addition to metformin, 28 days washout period will be performed before the initial screening	49 patients (25 for MEDI0382 and 24 for placebo)	MEDI0382 titrated up to 300 μg *N* = 25	*N* = 24 Participants received placebo matched to MEDI0382 dose	7 months
Parker (2020) [17]	Efficacy, safety, and mechanistic insights of cotadutide a dual receptor glucagon-like peptide-1 and glucagon agonist	RCT, Germany	• Patients aged ≥ 18 years had T2DM taking metformin monotherapy, • HbA1c ranged from 6.5–8.5% • BMI of 27–40 kg/m2 • Individuals receiving metformin monotherapy were eligible if no significant dose changes (increase or decrease ≥ 500 mg/day) occurred in the three months before screening • Patients receiving adjuncts to metformin were allowed after a 4-week washout period	65 patients (46 for MEDI0382 and 19 for placebo)	MEDI0382 titrated up to 300 μg *N* = 46	*N* = 19 Participants received placebo matched to MEDI0382 dose	6 months
NCT03550378	A Study to Look at the Effect MEDI0382 Has on Blood Sugar in People with Type 2 Diabetes and Kidney Problems and Also to Check That MEDI0382 is Well Tolerated	RCT, Germany, United Kingdom	• Patients aged ≥ 18 and < 85 years at the screening with T2DM managed with any insulin or oral therapy combination where no significant dose changes of oral therapy of more than 50% have occurred in the three months before screening • Body mass index (BMI) between 25 and 45 kg/m^2 • Haemoglobin A1c (HbA1c) range of 6.5% to 10.5% • eGFR ≥ 30 and < 60 mL/min/1.73 m^2 • Approximately 16 participants (40%) are required to have a screening eGFR ≥ 30 and < 45 mL/min/1.73 m^2, and at least 16 participants (40%) are required to have screening eGFR ≥ 45 and < 60 mL/min/1.73 m^2	41 patients (21 for MEDI0382 and 20 for placebo)	MEDI0382 titrated up to 300 μg *N* = 21	*N* = 20 Participants received placebo matched to MEDI0382 dose	7 months

*RCT* Randomized controlled trial, *N* Number, *RAI* Radioactive iodine therapy, *MBg* Megabecquerel, *mCi* Millicurie

**Table 2:** Baseline characteristics of enrolled patients in each included study. Data are expressed as mean and standard deviation (SD) or frequency and percentage

Study ID		Groups	Number of patients	Age mean ± SD	Males (%)	Body mass index (BMI) (kg/m2)	Weight (kg)	Race			
								**White**	**Black**	**Asian**	**American Indian or Alaska Native**
**P. Ambery et al. 2018 **[6] **(MAD study)**		MEDI0382 300 mcg	11	54.8 ± 6.8	7 (64%)	33.2 ± 4.2	99.7 ± 16.7	11 (100%)	0	0	0
		placebo	19	57.7 ± 6	12 (63%)	31.2 ± 3.1	88.5 ± 11.4	19 (100%)	0	0	0
**P. D. Ambery 2018 **[15]		MEDI0382 300 mcg	6	28.3 ± 5.9	6 (100%)	27.4 ± 2.8	-	6 (100%)	0	0	0
		placebo	12	32.8 ± 9.1	12 (100%)	25.7 ± 2.4	-	11 (91.7%)	0	0	0
**Rajaa Nahra 2021 **[16]		MEDI0382 300 mcg	256	56.3 ± 10.2	127 (94.6%)	-	-	252 (98.4%)	3 (1.2%)	1 (0.4%)	0
		placebo	112	57.3 ± 9.5	57 (50.9%)	-	-	107 (95.5%)	0	1 (0.9%)	3 (2.7%)
**NCT03645421**		MEDI0382 300 mcg	15	57.5 ± 9.2	13 (86.7%)	-	-	0	0	15 (100%)	0
		placebo	16	60 ± 8.6	13 (81%)	-	-	0	0	16 (100%)	0
**NCT03596177**		MEDI0382 300 mcg	19	59.5 ± 8.4	18 (94.7%)	-	-	18 (94.7%)	0	0	0
		placebo	9	65.2 ± 7.2	7 (77.8%)	-	-	8 (88.9%)	1 (11.1%)	0	0
**NCT03444584**		MEDI0382 300 mcg	25	61 ± 8.2	15 (60%)	-	-	25 (100%)	0	0	0
		placebo	24	58.4 ± 10	12 (50%)	-	-	24 (100%)	0	0	0
**Parker 2020 **[17]	**Cohort 1**	MEDI0382 300 mcg	26	58.7 ± 8.5	19 (73%)	31.5 ± 3.5	95.6 ± 17.2	25 (96%)	0	0	0
		placebo	13	-	9 (69%)	31.6 ± 3.8	93.8 ± 21	13 (100%)	0	0	0
	**Cohort 2**	MEDI0382 300 mcg	20	61.9 ± 6	10 (50%)	31.1 ± 3.5	92.4 ± 8.7	20 (100%)	0	0	0
		placebo	6	-	5 (83%)	31.2 ± 3.5	93.7 ± 9.6	6 (100%)	0	0	0
**NCT03550378**		MEDI0382 300 mcg	21	71.1 ± 7.4	12 (57.1%)	-	-	20 (95.2%)	0	0	0
		placebo	20	70.9 ± 4.7	9 (45%)	-	-	20 (100%)	0	0	0

### Quality assessment results

**Fig. 2:**
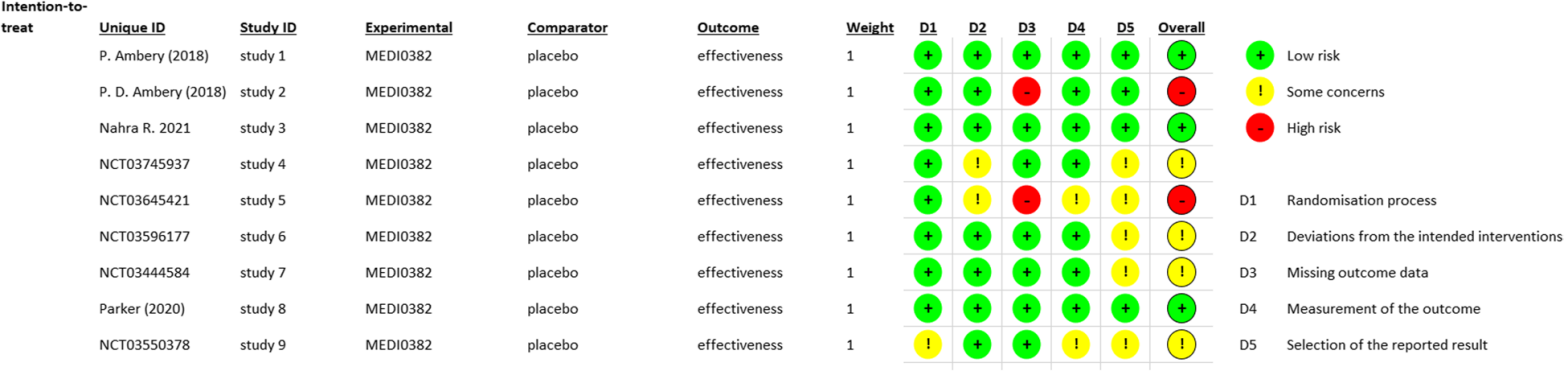
Risk of bias graph for randomized controlled trials using Excel tool to implement Rob2

**Fig. 3:**
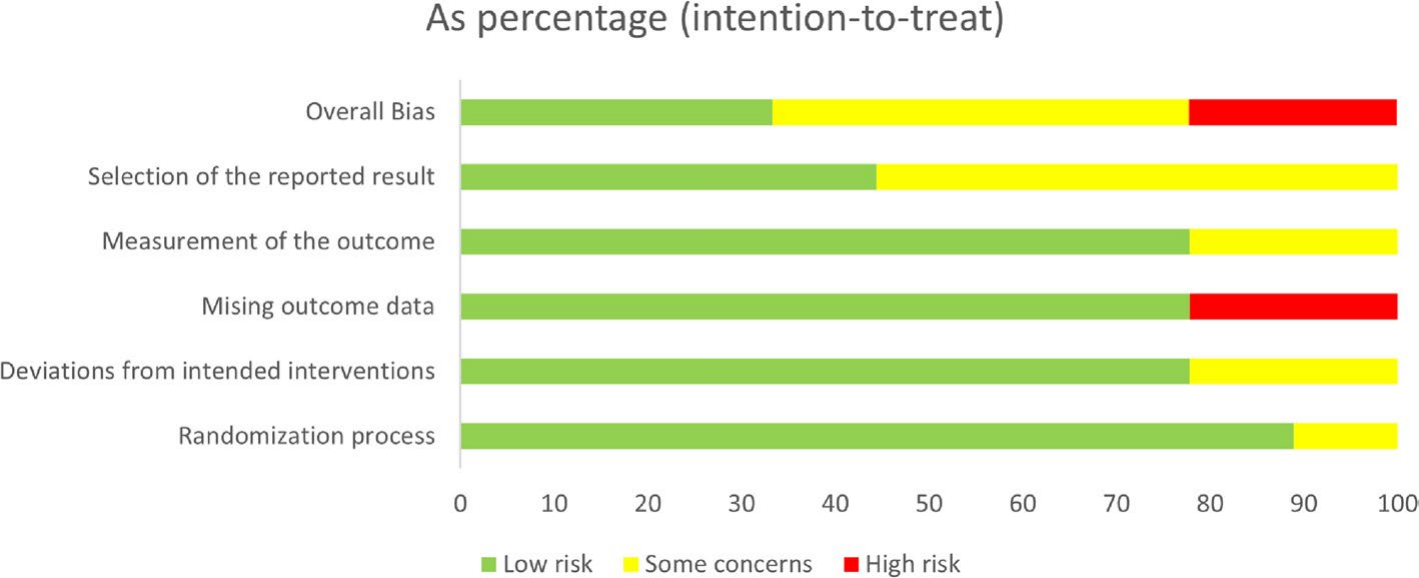
Risk of bias summary for randomized controlled trials using Excel tool to implement Rob2

The risk of bias summary is illustrated in Figs. 2 and 3. Regarding the Randomization process bias, all the studies were of low risk in terms of the randomization process except for NCT03550378, which was judged as some concerns because there was inadequate information about the allocation concealment, randomization, and baseline balance.

Regarding the intended interventions bias, most of the included trials had a low risk of bias in terms of deviations from the intended interventions except for NCT03645421 and NCT03745937, which were judged as some concerns. This is because there was no information about the statistical analysis used to estimate the effect of assignments in both of them despite blinding the personnel.

Regarding the missing outcome data bias, most of the included trials had a low risk of bias in terms of the missing outcome data due to applying the intention to treat analysis. We judged NCT03645421 and P.D. Ambery et al. as high risk of bias because the authors applied as-treated analysis [15].

Regarding the measurement outcome bias, we judged the risk of bias in the measurement of the outcome as low risk of bias in most of the studies due to blinding of all outcome assessors and using appropriate methods in measuring the outcomes. We judged NCT03645421 and NCT03550378 as some concerns due to the lack of information about blinding the outcome assessor.

For the selection of the reported results bias, the risk of bias due to the selection of the reported results ranged between low and some concerns. We judged all the registered protocols as some concerns because there is no published data yet to compare it with the protocols. The published studies [6, 15–17] were of low risk as all outcomes mentioned in the results were present in the protocols.

For other sourced of bias, we judged almost all the studies as high risk in terms of other potential sources of bias as most of them are registered protocols without any published papers yet. Parker et al. [17] stated the lack of statistical power to draw inferences between cohorts and the absence of validated questionnaires as a limitation in their study and so we judged it as having a high risk of bias. Accordingly, Ambery et al. [6] had a relatively small population size which we considered as high-risk potential. Only [15, 16] showed no other potential sources of bias.

### Efficacy endpoints

#### Percentage decrease in body weight

**Fig. 4:**
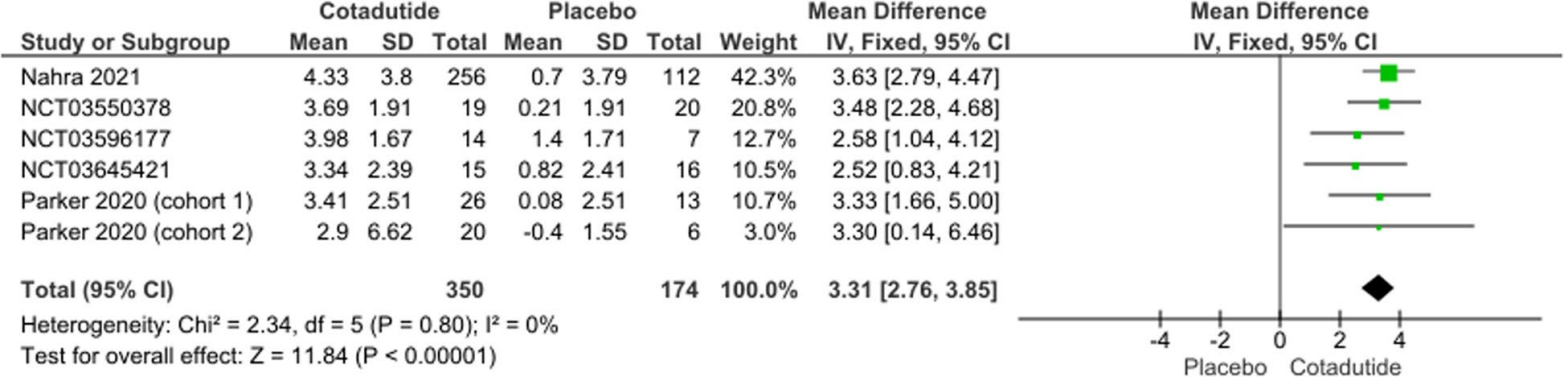
Percentage decrease in body weight plot

The pooled effect estimates of five studies favored Cotadutide 300 mcg over placebo (MD = 3.31, 95% CI [2.76, 3.38], *p* > 0.00001). Pooled data were homogenous under a fixed effect model (*p* = 0.80, I^2^ = 0%); Fig. 4.

#### Decrease in glycated hemoglobin (HbA1c)

**Fig. 5:**
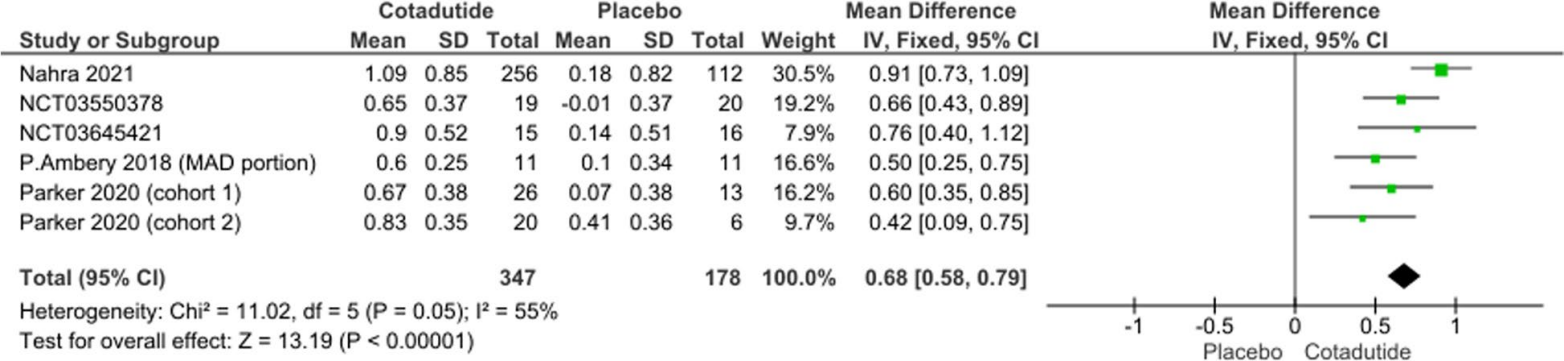
Decrease from baseline in Glycated Haemoglobin (HbA_1c_) plot

The pooled effect estimate of five studies showed that Cotadutide is significantly better than placebo (MD = 0.68, 95% CI [0.58, 0.79], *p* > 0.00001). Pooled data were homogenous under a fixed effect model (*p* = 0.05, I^2^ = 55%); Fig. 5.

#### Percentage decrease in glucose area under the plasma concentration curve (AUC [0-4 h])

**Fig. 6:**
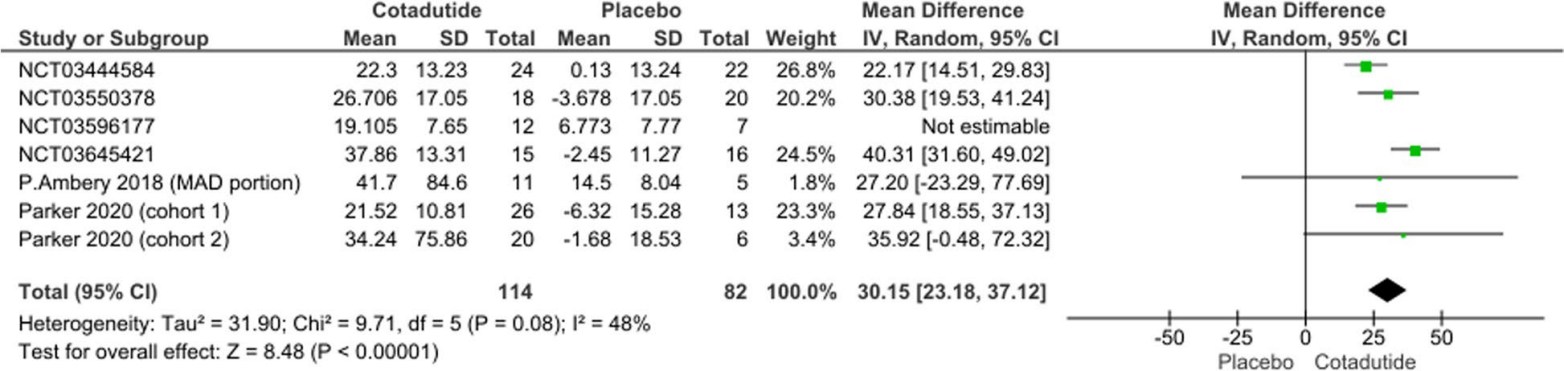
percentage decrease in glucose area under the plasma concentration curve (AUC [0-4 h]) plot

The pooled effect estimates of six studies favored Cotadutide 300 mcg over placebo (MD = 30.15, 95% CI [23.18, 37.12], *p* > 0.00001). Pooled data were heterogeneous (*p* = 0.0002, I^2^ = 77%) under a random effect model and the heterogeneity was best resolved by leaving out NCT03596177 (*p* = 0.08, I^2^ = 48%); Fig. 6.

#### Decrease from baseline in fasting plasma glucose over time (mg/dl)

**Fig. 7:**
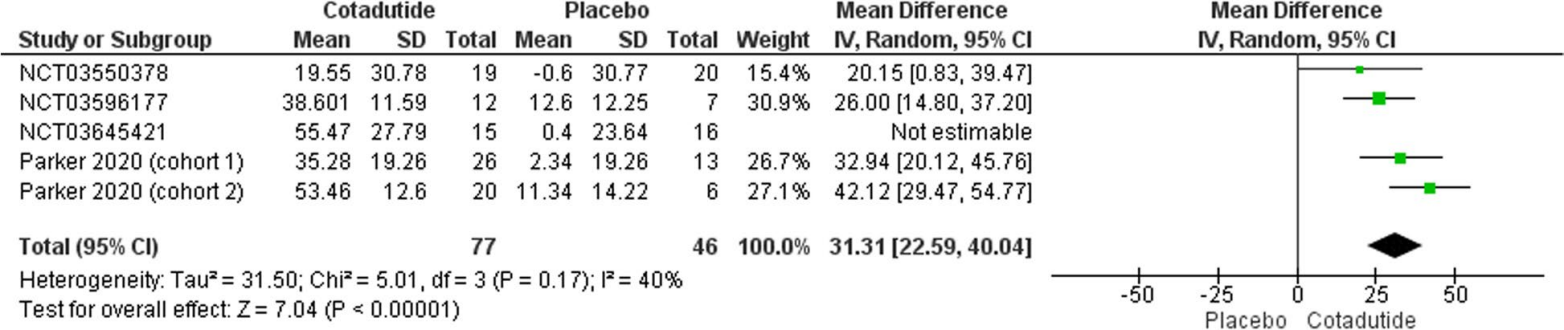
Change from Baseline in Fasting Plasma Glucose over Time (mg/dl) plot

The pooled effect estimate of four studies favored Cotadutide over the placebo (MD = 31.31, 95% CI [22.59, 40.04], *p* > 0.00001). Pooled data were heterogeneous (*p* = 0.03, I^2^ = 63%) under a random effect model and the heterogeneity was best resolved by leaving out NCT03645421 (*p* < 0.17, I^2^ = 40%); Fig. 7.

#### Anti-drug antibodies (ADA)

Nahra et al. [16] reported a statistically significant increase in the number of participants with ADA in the Cotadutide group over the placebo (155 out of 256 in the Cotadutide group and three out of 256 in the placebo group); NCT03444584, NCT03550378, and NCT03596177 reported non-significant results on the number of participants having ADA. NCT03444584 reported 2 out of 24 and 1 out of 24 in the Cotadutide group and the placebo group, respectively. In NCT03550378, two out of 21 participants in the Cotadutide group had ADA in comparison to the 20 persons on placebo in which none of them developed ADA. In NCT03596177, three out of 14 and zero out of seven participants experienced ADA in the Cotadutide group and the placebo group, respectively.

### Safety endpoints

#### Treatment-emergent adverse events (TEAEs)

**Fig. 8:**
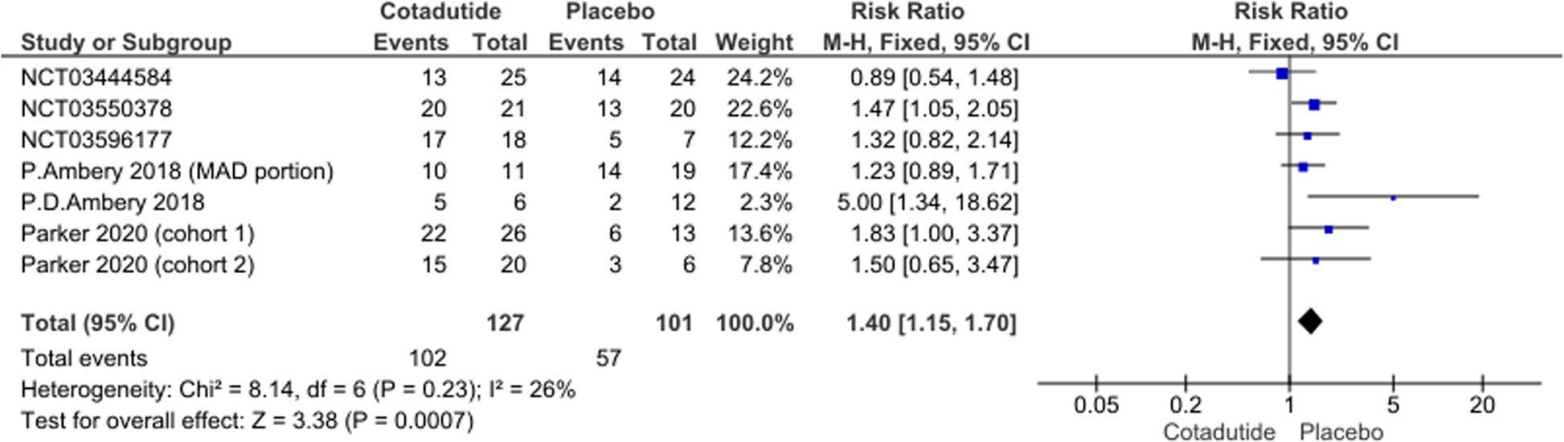
Treatment-Emergent Adverse Events (TEAEs) plot

The pooled effect estimate of six studies showed a statistically significant increased risk of TEAEs in the Cotadutide group compared to placebo (RR = 1.40, 95% CI [1.15, 1.70], *p* = 0.0007). Pooled data were homogenous under a fixed-effect model (*p* = 0.23, I^2^ = 26%); Fig. 8.

#### Treatment-emergent serious adverse events (TESAEs)

We didn’t do a meta-analysis for this outcome because none of the participants suffered any TESAEs in three out of six studies in both the Cotadutide and the placebo group. Only three studies reported some participants having TESAEs and they are relatively very low. In terms of TESAEs, NCT03550378 reported two out of 21 persons in the Cotadutide group and two out of 20 in the placebo group. On the other hand, NCT03596177 reported two persons out of 18 and zero out of seven in the Cotadutide group and the placebo group, respectively. In the MAD portion of the study by P. Ambery et al., they reported one out of seven participants having TESAEs in the Cotadutide 200 mcg group and none out of 19 participants in the placebo group [6].

## Discussion

This meta-analysis on 1258 participants with type 2 diabetes revealed that efficacy outcomes, including body weight, fasting blood glucose, HbA_1c_, and AUC [0-4 h], were significantly better in people receiving Cotadutide treatment than placebo. The number of participants with positive ADA to Cotadutide was high but without a significant difference compared to placebo. Furthermore, no significant difference was observed between the Cotadutide group and placebo in TESAEs. Hence, Cotadutide is safe and effective as a hypoglycemic drug in people with controlled type 2 diabetes.

In ten years, more than half of individuals with type 2 diabetes mellitus switch from oral monotherapy (usually Metformin) to insulin therapy to control their blood glucose levels [18]. Multiple combination therapies are routinely used before insulin is initiated. Insulin use causes weight gain, which can exceed 6 kg 20 in the first year after starting insulin medication [19]. The overall gain in weight can cause an increase in insulin resistance which is associated with high blood pressure, dyslipidemia, and a high risk of cardiovascular mortalities and morbidities such as non-fatal myocardial infarction or stroke, both before and after diagnosis of diabetes [20, 21]. Pre-clinical findings further suggest that the balance of activities at GLP-1 receptors and glucagon receptors was appropriate for both weight reduction and glycemic management [22]. These activities are supposed to be balanced by stimulating insulin release mediated by glucose, delayed gastric emptying, and enhanced oxidation of fatty acids [23, 24]. GLP-1, including Cotadutide (MEDI0382) and glucagon receptor dual agonists, may have central impacts on appetite as glucagon receptor agonist has been found in animal and human studies to increase energy expenditure [25].

Cotadutide (5–300 µg) corrected the glucose levels to the normal range in phase one of the first human trial which was conducted on healthy volunteers, with a pharmacokinetic profile that included once-daily treatment [6]. Similar to these previous findings [6, 15], in phase 2a, Cotadutide (100–300 μg) significantly lowered blood glucose levels and body weight indices in overweight or obese Japanese people with type 2 diabetes throughout a 48-day treatment period compared to placebo. Parker et al. found a substantial decline in glucose AUC (0-4 h) by − 21.52% with up titrated Cotadutide (50–300 μg) in comparison to + 6.32% with placebo. Similarly, a decline in body weight was reported by − 3.41% versus − 0.08% for Cotadutide versus placebo, respectively [17]. Nevertheless, in different study [26], with a lower BMI (26.3–28.8 kg/m2) than Parker et al. (31.5 kg/m2) [17], blood glucose and weight reduction with Cotadutide 300 μg remained significant compared to placebo at − 37.86% versus + 2.45% and − 3.34% versus − 0.82%, respectively.

Cotadutide therapy reduced body weight in a dose-dependent approach, and the highest reductions occurred at 300 μg. Moreover, Cotadutide improved fasting plasma glucose, fructosamine, HbA_1c_, percentage of time in hyperglycemia, insulin secretion, and resistance. After 6 weeks of Cotadutide medication, significant decreases in HbA_1c_ were found, with efficacy remaining constant [26].

Cotadutide has also been known to significantly reduce hepatic glycogen and steatosis, as well as having a beneficial effect on hepatic inflammation and fibrosis markers [26, 27]. The decrease in hepatic glycogen contrasts with what would be expected from a GLP-1 mono-agonist, which would cause glycogen accumulation and exhibit glucagon receptor interaction [28]. Furthermore, the degree of liver fat loss with Cotadutide (39% reduction) was comparable to that shown in a small study of women three months following bariatric surgery (42% reduction) [29]. This decrease in liver fat found with Cotadutide was larger than would be expected from weight loss alone—for example, in individuals with documented non-alcoholic fatty liver disease, a 5% decrease in BMI results in a 25.5 percent relative decline in liver fat [30].

MEDI0382 had a linear pharmacokinetic profile in the first human study on healthy volunteers (phase 1), and no participants tested positive for ADA [15]. In a previous study, participants were given Cotadutide for a year and had a significant ADA incidence. Only 16% of participants acquired ADAs over a titer of 80, at which point the influence on pharmacokinetics was around two times higher than the population average [16] (ClinicalTrials.gov identifier NCT04019561).

In the Harmony Outcomes study, albiglutide outperformed placebo in terms of serious adverse cardiovascular problems in people with type 2 diabetes and cardiovascular morbidities with a hazard ratio of 0.78, which implies that GLP-1 agonists can improve cardiovascular outcomes according to these data [31]. Due to GLP-1 and glucagon receptor agonism on the heart and vascular system, an increase in heart rate was expected. The rise in heart rate by 6.8 beats per minute observed with Cotadutide was not significantly greater than that seen with the GLP-1 receptor agonist liraglutide which increased by 6 to 9 beats from baseline. Furthermore, the drop in blood pressure was comparable to that seen with GLP-1 receptor agonists [23, 32].

Cotadutide plasma concentrations increased in agreement with the anticipated dose titration at all dose levels, with no TEAEs linked to immunogenicity observed [6]. In this study, Cotadutide had a higher rate of gastrointestinal co-morbidities such as nausea and vomiting compared to placebo. This outcome is also seen with the GLP-1 receptor mono-agonists [33, 34]. In addition, Cotadutide's safety profile was equivalent to that of previous global trials [6, 15], with a greater incidence of gastrointestinal adverse events.

To lower the gastrointestinal adverse events associated with Cotadutide 300 μg, dose escalation was required upon which a phase 2 study in obese type 2 diabetes participants reported that Cotadutide was effective and well-tolerated with starting doses of 50 μg for 7 days, then gradual dose escalation up to 300 μg [17, 35]. Despite causing more gastrointestinal upset than placebo, escalated dosages of Cotadutide of up to 300 μg which were given once daily were generally tolerated because the symptoms were mild or moderate in severity [15].

This study comprehensively evaluated the efficacy and safety of Cotadutide for people with type 2 diabetes. Nine RCTs were included in the study, resulting in a valuable evidence level. The included trials varied from low to high quality. The majority of the identified heterogeneity was resolved. Our analysis also has certain limitations, including the small sample size and the small number of included studies. We faced some limitations in our study, which include the following. Publication bias could not be detected due to the small number of included studies. Exclusion of studies published in the non-English language. The short follow-up period and lack of placebo were the major drawbacks of the study. Most of the included studies were protocols with published results, not articles. Cotadutide medication should also be evaluated for its effects on stomach emptying, energy intake, and energy expenditure in larger studies.

## Conclusions

Over a short dosage period, Cotadutide provided considerable metabolic benefits to overweight and obese participants with type 2 diabetes. Cotadutide’s safety and pharmacokinetics allow once-daily administration of dosages less than 150 μg, which can be followed by dose escalation. Cotadutide's promising impacts on glycemic control, body weight, and liver fat suggest that it might be a helpful agent for type 2 diabetes individuals with longer-term treatment.

## Supplementary Information

**Additional file 1.**

## Data Availability

All data generated or analyzed during this study are included in this published article or in the data repositories listed in References.

## Abbreviations

MD: Mean difference
HbA_1c_: Glycated hemoglobin
IDF: International Diabetes Federation
GLP-1: Glucagon-like peptide-1
PRISMA: Preferred Reporting Items for Systematic Reviews and Meta-Analysis
CENTRAL: Cochrane Central Register of Controlled Trials
RCT: Randomized Controlled Trials
RoB 2: Cochrane risk-of-bias tool for randomized trials
TEAEs: Treatment-Emergent Adverse Events
RR: Risk Ratio
CI: Confidence Intervals
AUC [0-4 h]: Percentage decrease in glucose area under the plasma concentration curve
ADA: Anti-drug antibodies
TEAEs: Treatment-emergent adverse events
TESAEs: Treatment-emergent serious adverse events
