# Supplementary material for: Impact of Cotadutide drug on patients with type 2 diabetes mellitus: a systematic review and meta-analysis
Source: BMC Endocr Disord. 2022 Apr 29;22:113. doi: 10.1186/s12902-022-01031-5 (PMC9055739; doi:10.1186/s12902-022-01031-5)
Supplement: Supplementary file 1 — Additional file 1. [file 12902_2022_1031_MOESM1_ESM.docx]

**Impact of Cotadutide Drug on Obese Individuals with Type 2 Diabetes Mellitus: A Systematic Review and Meta-analysis**

**Mahmoud M Ali^1*^, Ahmed Hafez^2^, Mahmoud Shaban Abdelgalil^3^, Mohammed Tarek Hasan^4^, Mohammed Magdy El-Ghannam^5^, Osama M Ghogar^6^, Asmaa Ahmed Elrashedy^7^, Mohamed Abd-ElGawad^8^**

1. *Faculty of Pharmacy, Al-Azhar University, Assiut, Egypt.*

[*MahmoudEbrahim.4.2045@azhar.edu.eg*](mailto:MahmoudEbrahim.4.2045@azhar.edu.eg)

1. *Faculty of Medicine, Menoufia University, Shebin El-Kom, Menoufia, Egypt.*

[*ahmedhafez21@med.menofia.edu.eg*](mailto:ahmedhafez21@med.menofia.edu.eg)

1. *Faculty of Medicine, Ain-shams University, Cairo, Egypt.*

[*29908068800596@med.asu.edu.eg*](mailto:29908068800596@med.asu.edu.eg)

1. *Faculty of Medicine, Al-Azhar University, Cairo, Egypt.*

[*MohamedTarek.med.stu.2@azhar.edu.eg*](mailto:MohamedTarek.med.stu.2@azhar.edu.eg)

1. *Faculty of Pharmacy, German University in Cairo, Egypt*

[*mahammed.zaki97@gmail.com*](mailto:mahammed.zaki97@gmail.com)

1. *Faculty of Pharmacy, Al-Azhar University, Assiut, Egypt.*

[*OsamaMagdy.2020@azhar.edu.eg*](mailto:OsamaMagdy.2020@azhar.edu.eg)

1. *Faculty of Medicine, Kafr El-Shaikh University, Kafr El-Shaikh, Egypt.*

[*asmaa.ahmed.4999@gmail.com*](mailto:asmaa.ahmed.4999@gmail.com)

1. *Faculty of Medicine, Fayoum University, Fayoum, Egypt.*

[*mohammed.mahmod87@gmail.com*](mailto:mohammed.mahmod87@gmail.com)

**Corresponding author*:**

Faculty of Medicine, Fayoum University, Fayoum, Egypt.

Postal address; 5 Al-Touba Street, from Al-Fanya Street, Al-Hadka road, Fayoum, Fayoum, Egypt

Tel. /Fax: +201069401202

Email: [mohammed.mahmod87@gmail.com](mailto:mohammed.mahmod87@gmail.com)

ORCID ID: 0000-0003-4120-9161

**Search strategy: -**

**PubMed:**

(Cotadutide OR MEDI0382 OR dual GIP/GLP-1 receptor agonist OR dual incretin) AND (Type 2 Diabetes Mellitus OR NIDDM OR Diabetes OR Diabetes Mellitus OR Non-Insulin-Dependant Diabete OR DM OR hyperglycemia OR glycemic control) AND (obes* OR body fat OR high BMI OR adiposity OR heaviness OR overweight OR weight loss OR BMI OR "waist-hip ratio" OR fat loss OR body mass index OR body weight)

No limitations were applied

Date of search: From 1 January 1979 to 1 June 2021

Results: 228

**Cochrane:**

1. Cotadutide OR MEDI0382 OR dual GIP/GLP-1 receptor agonist OR dual incretin
2. Type 2 Diabetes Mellitus OR NIDDM OR Diabetes OR Diabetes Mellitus OR Non-Insulin-Dependant Diabete OR DM OR hyperglycemia OR glycemic control
3. obes* OR body fat OR high BMI OR adiposity OR heaviness OR overweight OR weight loss OR BMI OR "waist-hip ratio" OR fat loss OR body mass index OR body weight
4. #1 AND #2 AND #3

No limitations were applied

Results: 64

**Scopus:**

ALL((Cotadutide OR MEDI0382 OR “dual GIP/GLP-1 receptor agonist” OR “dual incretin”) AND (“Type 2 Diabetes Mellitus” OR NIDDM OR Diabetes OR “Diabetes Mellitus” OR “Non-Insulin-Dependant Diabete” OR DM OR hyperglycemia OR “glycemic control”) AND (obese OR “body fat” OR “high BMI” OR adiposity OR heaviness OR overweight OR “weight loss” OR BMI OR "waist-hip ratio" OR “fat loss” OR “body mass index” OR “body weight”))

No limitations were applied

Results: 345

**Web of Science:**

TS=((Cotadutide OR MEDI0382 OR “dual GIP/GLP-1 receptor agonist” OR “dual incretin”) AND (“Type 2 Diabetes Mellitus” OR NIDDM OR Diabetes OR “Diabetes Mellitus” OR “Non-Insulin-Dependant Diabete” OR DM OR hyperglycemia OR “glycemic control”) AND (obes* OR “body fat” OR “high BMI” OR adiposity OR heaviness OR overweight OR “weight loss” OR BMI OR "waist-hip ratio" OR “fat loss” OR “body mass index” OR “body weight”)

No limitations were applied

Results: 26
